# Changes in digital healthcare search behavior during the early months of the COVID-19 pandemic: A study of six English-speaking countries

**DOI:** 10.1371/journal.pdig.0000241

**Published:** 2023-05-01

**Authors:** Robin van Kessel, Ilias Kyriopoulos, Alicja Mastylak, Elias Mossialos

**Affiliations:** 1 LSE Health, Department of Health Policy, London School of Economics and Political Science, London, United Kingdom; 2 Department of International Health, Care and Public Health Research Institute (CAPHRI), Maastricht University, The Netherlands; 3 Gravitate Health, European Patients’ Forum, Brussels, Belgium; 4 Duke Clinical Research Institute, Duke University School of Medicine, Durham, North Carolina; 5 Institute of Global Health Innovation, Imperial College London, London, United Kingdom; Iran University of Medical Sciences, IRAN (ISLAMIC REPUBLIC OF)

## Abstract

Public interest is an important component influencing the likelihood of successfully implementing digital healthcare. The onset of the COVID-19 pandemic allowed us to assess how public interest in digital health changed in response to disruptions in traditional health services. In this study, we used a difference-in-differences approach to determine how digital healthcare search behavior shifted during the early months of the COVID-19 pandemic compared to the same period in 2019 across six English-speaking countries: the United States, Canada, the United Kingdom, New Zealand, Australia, and Ireland. In most cases, we observed that the official declaration of the COVID-19 pandemic on 11 March 2020 was associated with a significant overall increase in the volume of digital healthcare searches. We also found notable heterogeneity between countries in terms of the keywords that were used to search for digital healthcare, which could be explained by linguistic differences across countries or the different national digital health landscapes. Since online searches could be an initial step in the pathway to accessing health services, future studies should investigate under what circumstances increased public interest translates into demand for and utilization of digital healthcare.

## Author summary

It is important to understand how people behave when looking to implement new technologies, such as digital healthcare. The COVID-19 pandemic caused a large change in how healthcare was able to be organized and delivered, which means people were required to change as well. In this study, we investigated whether the pandemic changed the online search behavior for digital healthcare. We included six English-speaking countries: the United States, Canada, the United Kingdom, New Zealand, Australia, and Ireland. In most cases, digital health searches increased because of the pandemic. We saw that the keywords used to search for digital health differed per country, which indicates differences in language preferences and national digital health systems. Because online searches can form the first step in the process of accessing health services, further work should explore when an increase in public interest (as captured in this article) translates into actual use of digital healthcare.

## 1. Introduction

The COVID-19 pandemic has greatly impacted the use of digital healthcare and access to traditional healthcare services around the world [1,2]. The pandemic resulted in a shift towards the use of telemedicine and other digital healthcare services, as people sought to reduce the risk of infection by avoiding in-person visits to hospitals and clinics or health services were deliberately restricted to remote delivery [3,4]. Simultaneously, the pandemic has widely disrupted traditional healthcare systems, leading to shortages of personal protective equipment and other supplies, as well as challenges in delivering care to patients [1]. Countries have also shown a variety of responses to the pandemic [5]. Those with a more digitally mature infrastructure and health systems were able to more easily adapt to the challenges posed by COVID-19 [2,6]. Nevertheless, concerns regarding the fitness of digital health-oriented policies and their ongoing roles in the health ecosystem remain [2,6,7].

Appropriate implementation of these new policies will require an improved understanding of whether members of the general population are interested in seeking access to digital healthcare [8]. Overall, online search volumes for healthcare-related topics have increased significantly over the course of the pandemic [9,10]. While search engines cannot substitute health professionals as a sole health information source due to a combination of the prominence of misinformation and a lack of health, digital, and science literacy, [11,12] search engine data can be instrumental in understanding the general preference of populations in exploring the possibility of using digital health [13,14]. It is unclear whether search behavior is always translated into actual demand for healthcare services, but evidence suggests that online searches could be an initial pathway of seeking access to health services [13–15].

The onset of the COVID-19 pandemic created a natural experiment that permitted us to determine whether and how health-seeking behavior changed in response to disruptions in traditional services [16,17]. Previous research using interrupted time-series indicated that digital health search behavior spiked during the early stages of the pandemic, though it declined shortly afterwards [15]. However, this research was only able to identify the structural breaks within a time-series and did not allow for control groups in the study design. In this study, we hypothesized that individuals became more interested in digital healthcare during the early months of the COVID-19 pandemic compared to the same period in 2019. To test this hypothesis, we quantified Google search volumes related to healthcare and compared findings from before to those collected during the first few months after the announcement of the pandemic as a proxy for digital healthcare-seeking behavior [13,15,18]. Evaluation of data collected from Google Trends can be used to understand, monitor, and potentially forecast public interest and information-seeking trends and has become an increasingly popular method for assessing population preferences in various fields associated with health research [14,19,20].

## 2. Methods

This article has a quasi-experimental study design and uses a difference-in-difference (DiD) analysis to estimate the change in public search behavior in digital health between 2019 and 2020 [21]. The design used for this analysis differed somewhat from conventional DiD regression techniques. Specifically, we followed methodological adaptations [22,23], which have been also used in previous research of Google Trends [24] and are discussed step-wise below.

### 2.1. Google Trends and data access

Google Trends is the principal tool used to study trends and patterns of search engine queries using Google [14]. It provides both current and archived information on Google queries from 2004 onwards. Data are available in real-time, solving issues that arise with conventional and more time-consuming survey methods [25]. Raw search volumes are normalized, scaled, and presented in a range from 0 to 100 points to provide quantitative information on the volumes of specific searches relative to the total number of Google searches. The algorithm used to respond to these queries adjusts relative search volumes based on internet access and population size. While daily search volume data can be provided for <9 months, weekly data are provided for time periods between 9 months and 5 years. Thus, Google Trends has been used to examine population preferences in various disciplines, including mental health, oncology, ophthalmology, and economics [24,26,27]. Daily data for Google Trends were extracted for 1 February 2019–1 August 2019 and 1 February 2020–1 August 2020, as well as weekly Google Trends data for 1 February 2019–1 August 2020 were downloaded on 23 May 2022. Data on COVID-19 cases and deaths were also retrieved on 23 May 2022 from the Oxford COVID-19 Government Response Tracker, which contains daily updated COVID-19 epidemiological and policy data for more than 180 countries [28]. This study did not require independent review board approval or patient informed consent. This study followed the methodological framework for use of Google Trends data in infodemiology and infoveillance as well as the Strengthening the Reporting of Observational Studies in Epidemiology (STROBE) reporting guidelines [25].

### 2.2. Country, timeframe, and keyword selection

The United States, Canada, the United Kingdom, New Zealand, Australia, and Ireland were chosen as subjects for this study because they share English as a dominant language and because 90–97% of the population in each of these countries has access to the internet [29]. These countries were in different stages of development with respect to the integration of digital health both before and during the early stages of the pandemic [6,30]. Google is used for 87–93% of the online search queries in each of these countries [31–33]; this suggests that our findings will accurately capture the population search behavior in each country under study [19].

Data were extracted from February 1 to August 1 in both 2019 and 2020 to ensure that our collection was sufficient for time points both before and after the official declaration of the pandemic by the World Health Organization (WHO) on 11 March 2020. We collected daily Google Trends data for five specific keywords, which were selected from digital health search strings in recent literature reviews [34–36]: *online doctor*, *telehealth*, *online health*, *telemedicine*, and *health app*. The combination of these keywords allowed us to capture the public interest across different types of digital health (e.g., video consultations, telemedicine, and healthcare delivered through mobile apps). While we also considered the keywords *digital health*, *digital therapeutics*, *telecare*, *teleconsultation*, *telesupport*, *mhealth*, *mobile health*, *telemonitoring*, and *virtual health*, no data on search volumes was available for this second set of keywords. Thus, we did not include the results of these searches in our analysis. All keyword assessments were performed on a country-by-country basis using the Google Trends online interface. The term *eHealth* was also excluded as it tends to be used primarily in academic settings and is not expected to be a term used when people seek for digital health services. Despite the use of common terms (i.e., *online doctor* and *online health* or *online health* and *health app*), we avoided obtaining duplicate search results across multiple keywords by including relevant quotation marks in our data extraction commands (e.g., “online doctor”).

We collected daily Google Trends search data that covered two six-month periods, between 1 February and 1 August in both 2019 and 2020. Because the daily data for these two independent six-month periods in 2019 and 2020 were collected in two separate requests, the scaling factors used by Google Trends to calculate the relative search volumes were unlikely to be the same. Therefore, we needed to rescale the two series so the findings will be comparable to one another. The scaling procedure used to normalize these data has been reported extensively in a previous publication [24]. Similar to previous research of Google Trends during the COVID-19 pandemic [15,24], we assigned the events occurring during the time period before a cut-off date (e.g., 12 March 2020) as the control group, while those occurring thereafter are included as the test group. As such, we compared the search volumes taking place both pre- and post-pandemic announcement in 2020 with those performed before and after the same date in 2019.

### 2.3. Statistical analysis

We used a DiD regression analysis with the adaptation described above to compare results in identical timeframes in 2019 and 2020. We accounted for fixed effects from each country, as well as those associated with a specific year, week, and day. We added daily reports of COVID-19 cases and deaths as covariates and weighted the analysis by population sizes of the studied countries per 1 January 2020 taken from Worldometer [37]. In this study, we fit our data to the following regression model:

$$Y_{c,t}=\beta_{0}+\beta_{1}\;PostMarch11_{c,t}+\beta_{2}\;Year_{t}\;+\beta_{3}\left(PostMarch11_{c,t}\;*\;Year_{t}\right)+\gamma X_{c,t}+\eta Z_{c,t}+\upsilon_{c}+\rho_{t}+\varepsilon_{c,t}$$

In this model, Y_*c*, *t*_ reflects the volume of digital health searches in country c on day t. *PostMarch11*_*c*, *t*_ is a binary variable that takes the value of one for events occurring after the WHO pandemic declaration on 11 March in both studied years, and zero for those occurring beforehand. *Year*_*t*_ represents the year of the declaration of the pandemic, which was 2020. To control for variables relating to the spread and severity of the COVID-19 pandemic at any given time in each studied country as well as the heterogenous spread of COVID-19 across the studied countries, we also included *Χ*_*c*, *t*_ and ηZ_*c*, *t*_ that capture the seven-day moving average of daily reported COVID-19 cases and deaths, respectively. The symbols υ_*c*_ and ρ_*t*_ represent the country dummies and year, week, and day-level dummies, respectively. These account for the fact that spread of public information of the pandemic differed between countries. These are added as dummies under the assumption that the country-level confounders do not vary over time and the temporal confounders do not vary across countries, which mirrors the assumptions of previous Google Trends research covering the COVID-19 pandemic [24]. Further rationale to adding these variables as dummies is found in the structure of Google Trends data [25], which is nationally aggregated daily data that represents the digitally connected population [15,38]. Finally, ε_*c*, *t*_ is the error term. Robust standard errors were reported that were clustered at the day level. The WHO pandemic declaration was chosen as cut-off point for its profound societal impact, including availability of healthcare, willingness to access healthcare, and increased health anxiety.

A key assumption in our study design was that these anticipatory effects were absent surrounding the WHO pandemic declaration that may have had an impact on digital healthcare-related searches during the months before the official announcement. In other words, we expected that, in the absence of the pandemic, Google search levels determined for time periods pre- and post-March 11, 2020 would be similar in 2020 to those performed in past years (i.e., the coefficient of interest β_3_ would be statistically insignificant). We empirically tested this assumption by performing identical analyses for data collected during the years 2016, 2017, and 2018. We further accounted for random daily variation in search volumes by performing a sensitivity analysis using 7-day moving averages of Google search volumes to smooth out short-term fluctuations and highlight longer-term trends or cycles (see S1 Appendix p.6). All analyses were performed in Stata/MP (version 17).

### 2.4. Ethical considerations

This study has no inherent ethical implications or considerations. No animals or human participants were involved in this research. No personal data were used in this research. Thus, we did not seek a review of our study design from an institutional review board.

## 3. Results

### 3.1. Descriptive results

**Fig 1:**
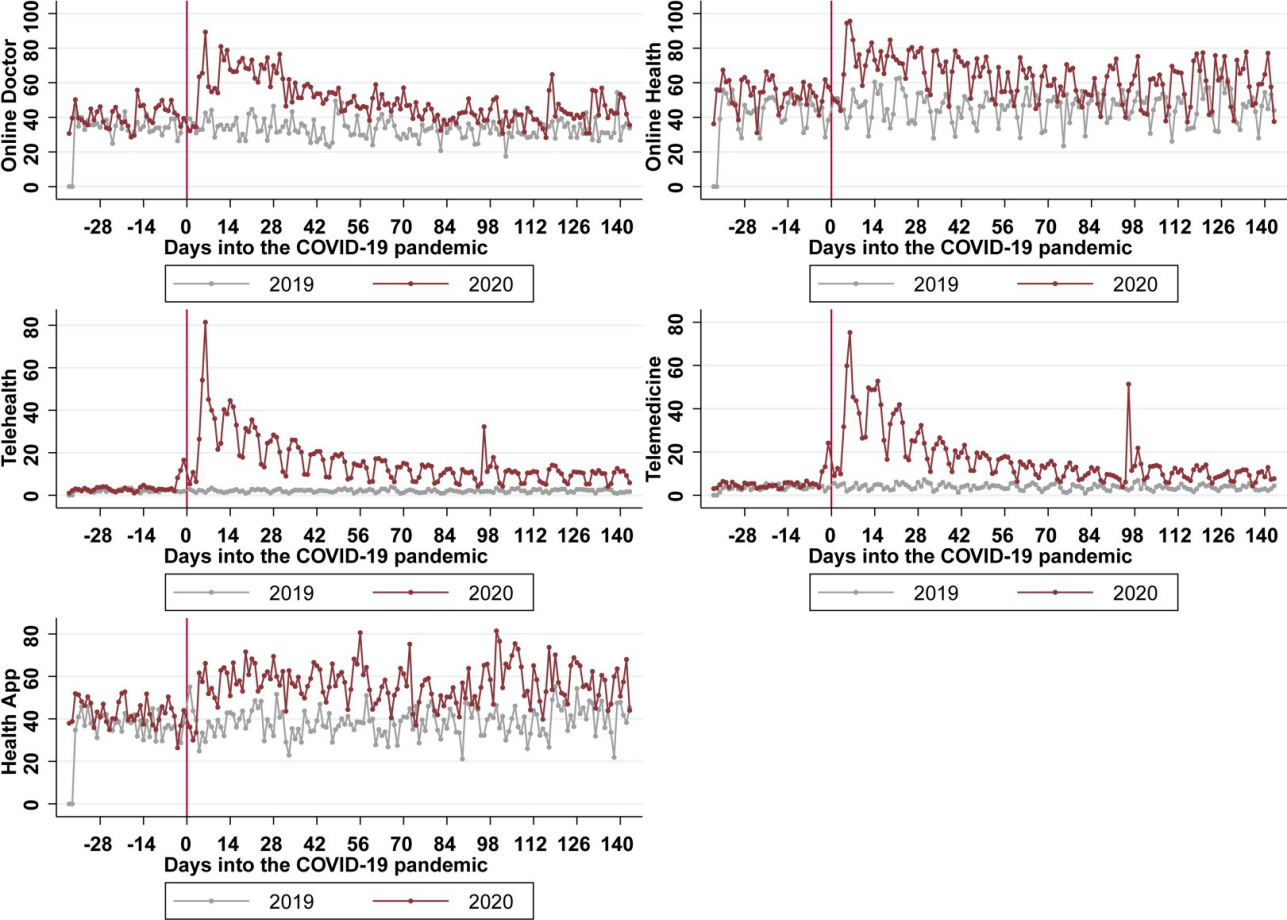
Google Trends scaled search volumes both before and after the WHO announcement of the COVID-19 pandemic on March 11, 2020, weighted by population size. The vertical axis documents the average search volume (scaled from 0 to 100) before and after March 11 (designated as Day 0) in both 2019 and 2020.

As shown in Fig 1, our findings weighted by population size revealed an overall increase in search volumes related to digital healthcare using each of the aforementioned five keywords after the announcement of the COVID-19 pandemic on 11 March 11 2020, compared to the same dates in 2019. Notably, *telehealth* and *telemedicine* seem to only be sought for after the pandemic announcement. The slight increases before the cut-off of 11 March 2020 can be attributed to the heterogenous spread of COVID-19 in the studied countries. Search volumes performed in each of the six countries under study are included in Figures A-F in S1 Appendix (pp. 3–5).

### 3.2. Difference-in-difference estimates

The official WHO announcement of the COVID-19 pandemic was associated with a significant increase in the overall intensity and volume of searches using digital health-related keywords (Table 1). Our findings revealed a 10.49-point increase (95% confidence interval [CI] 5.68–15.31) in the volume of searches performed using the keyword *telehealth*, an 8.54-point increase (95% CI 3.06–14.02) in the volume of searches featuring the keyword *telemedicine*, and a 7.03-point increase (95% CI 2.39–11.67) in the volume of searches that included the keyword *health app*. Our sensitivity analysis shows minor changes that can be attributable to the transformation of the variable-of-interest towards a moving average. In contrast to the main analysis, *online doctor* and *online health* were found to be significant in the sensitivity analysis. Additional details are included in Table B in S1 Appendix (p. 6).

**Table 1:** The effects of the pandemic announcement on digital health search volumes.

	Estimate	95% CI	P-value	Observations
Online doctor	3.83	-0.91–8.56	0.11	2172
Online health	4.65	-0.34–9.64	0.07	2172
Telehealth	10.49	5.68–15.31	< 0.001	2172
Telemedicine	8.54	3.06–14.02	< 0.01	2172
Health app	7.03	2.39–11.67	< 0.01	2172

Note: All models control for a dummy variable that takes the value of 1 in the days after the pandemic was announced. Country, year, week, and day of the week were included as dummies. Robust standard errors are used that are clustered at the day level.

The increases in search volumes based on specific keywords differed to some degree in each of the six countries evaluated (see Table 2 and Figure G in S1 Appendix). For example, in Australia, search volumes that included the terms *telehealth* and *health app* increased by 9.05 points (95% CI 4.25–13.85) and 9.85 points (95% CI, 0.82–8.87), respectively. Similarly, Canada reported a 16.12-point increase (95% CI 3.21–29.01) in the volume of searches using the keyword *online doctor*; the United Kingdom reported a similar increase of 13.23 points (95% CI 6.45–20.01) in searches using the keyword *health app*. The volume of searches performed in New Zealand featuring the keyword *telehealth* increased by 9.89 points (95% CI 3.30–16.48); those featuring the term *health app* increased by 11.19 points (95% CI 4.83–17.55). In the United States and Ireland, use of the term *telehealth* increased by 24.54 points (95% CI 13.90–35.19) and 8.80 points (95% CI 4.76–12.85), respectively; similarly, the use of the term *telemedicine* increased by 27.59 points (95% CI 15.50–39.68) and 5.52 points (95% CI 1.06–9.98), respectively in these two countries.

**Table 2:** The impact of the pandemic announcement on digital health search volumes in each country.

	**Australia**			**Canada**			**New Zealand**		
	**Coefficient**	**95% CI**	***p*-value**	**Coefficient**	**95% CI**	***p*-value**	**Coefficient**	**95% CI**	***p*-value**
**Online Doctor**	1.20	-13.33–15.71	0.87	16.12	3.21–29.01	0.02	-3.73	-18.66–11.20	0.62
**Online Health**	4.32	-4.92–13.57	0.36	0.33	-9.27–9.92	0.95	-1.73	-17.03–13.56	0.82
**Telehealth**	9.05	4.25–13.85	< 0.001	8.06	-4.78–20.90	0.22	9.89	3.30–16.48	< 0.01
**Telemedicine**	3.32	-1.28–7.92	0.16	5.31	-2.62–13.24	0.19	-0.51	-10.18–9.16	0.92
**Health App**	9.85	0.82–18.87	0.03	2.78	-8.18–13.74	0.62	11.19	4.83–17.55	< 0.01
	**United Kingdom**			**United States**			**Ireland**		
	**Coefficient**	**95% CI**	***p*-value**	**Coefficient**	**95% CI**	**p-value**	**Coefficient**	**95% CI**	***p*-value**
**Online Doctor**	-4.30	-9.81–1.21	0.13	6.28	-4.15–16.71	0.24	-6.95	-27.44–13.54	0.50
**Online Health**	-9.42	-18.83 –-0.02	0.05	4.20	-4.97–13.37	0.37	0.04	-8.97–9.06	0.99
**Telehealth**	3.07	-0.40–6.54	0.08	24.54	13.90–35.19	< 0.001	8.80	4.76–12.85	< 0.001
**Telemedicine**	1.56	-1.83–4.95	0.37	27.59	15.50–39.68	< 0.001	5.52	1.06–9.98	0.02
**Health App**	13.23	6.45–20.01	< 0.001	8.95	-0.76–18.67	0.07	10.70	-10.07–31.46	0.31

Note: All models control for a dummy variable that takes the value of 1 in the days after the pandemic was announced. Year, week, and day of the week were included as dummies. Robust standard errors are used that are clustered at the day level.

We tested our common trends assumptions by evaluating Google Trends data from 2016–2018. While a comparison of findings from 2016 and 2017 revealed no significant changes in search volumes featuring the keywords *online health*, *telehealth*, *telemedicine*, and *health app*, our findings revealed a significant decrease in search volumes featuring the keyword *online doctor* (-9.73 points; 95% CI -15.10 –-4.38). Similarly, no significant changes were reported in search volumes that included the keywords *online doctor*, *telehealth*, *telemedicine*, and *health app* in 2017 and 2018; by contrast, a significant reduction in searches featuring the keyword *online health* were reported during this period (-6.14 points; 95% CI -10.91 –-1.38). Additional details are included in Table A in S1 Appendix (p. 6).

## 4. Discussion

Our findings revealed an increase in the volume of searches that featured digital health-related keywords after 11 March 2020 compared to before and after controlling for the differences of the spread of disease. In particular, *telehealth* and *telemedicine* were only searched after the pandemic announcement, even though these terms have been in use since the early 2000’s [39,40], alluding to the possibility that these terms only came in common use during the pandemic. Some of the increases in search volume may be due to the disruption and unavailability of healthcare facilities and in-person services, increased health anxiety, or unwillingness to access in-person health services during the early months of the pandemic and the need to seek alternative sources of healthcare [2]; these findings may also reflect the nature of the healthcare system in each country. For example, the United States reported the largest increase in digital health search volumes, which aligns with previous research indicating a stark increase of digital healthcare utilization in the United States following the onset of the pandemic [41,42]. By contrast, Australia and New Zealand reported increases in search volumes using the keywords *telehealth* and *health app*; this may point toward the presence of a more established digital health ecosystem and the promotion of digital health services in these countries [43,44]. The United Kingdom also fits this profile; however, while we observed an increase in search volumes featuring the keyword *health app*, no significant changes were associated with the use of the keyword *telehealth*. The smaller increases observed in New Zealand can also be attributed to their effective pandemic response [45]. Even though these findings point towards some discrepancies in terminology across these six countries, our findings led us to conclude that the general public demonstrated an increased interest in digital healthcare during the early months of the COVID-19 pandemic.

The large increases in digital health search volumes specifically in the United States and the rise in the number of beneficiaries receiving digital healthcare early on in the pandemic may have been fueled by temporary policies and flexibilities that encouraged public and private payers to expand digital health coverage and reimbursement [46,47]. These findings suggest that the largest barrier to accessing digital health in the United States may be the policy landscape that prevents both prescription and reimbursement of this type of care. Unfortunately, the new policies were designed to be only temporary and may disappear once the pandemic officially ends. Although these policies remain in effect at the time of writing in December 2022, access to digital healthcare in the United States remains unstable. However, both Medicare and Medicaid services recently included certain forms of digital healthcare within their overall strategies; this important development may ultimately remove some of the structural barriers that prevent the full use of digital healthcare in the United States [47–50].

Interestingly, Australia and Canada took an approach that was similar to that used in the United States, and expanded medical benefits and services to accommodate digital health early during the pandemic [51,52]. However, the relative changes in digital health search volumes were far below those reported in the United States. In Australia, this was most likely because healthcare is generally affordable to all, and digital healthcare services had already been established and were in use prior to the pandemic [43]. Nevertheless, our results did reveal a significant increase in the search volumes that used the keyword *telehealth*, suggesting an overall increase in public interest in these services during the early months of the pandemic. In Canada, traditional healthcare services resumed as early as May 2020, which led to a decrease in digital health utilization from that point forward [52]. This may explain the overall smaller increases observed in search volumes reported in Canada. However, the increase in searches featuring the keyword *online doctor* remained significant even after this point in time, suggesting that some Canadians remained interested in accessing digital health services.

Our analysis of the common trends assumption revealed some fluctuations in digital search volumes from 2016 to 2018. However, the statistically significant fluctuations are exclusively those documenting decreases in search volumes; no significant increases were reported during this timeframe. These findings are in stark contrast to the results of our analysis of the pandemic period, in which significant increases in searches focused on *telehealth*, *telemedicine*, and *health app* were observed, thus suggesting that our analysis was not impacted by seasonal changes or artificial correlation. Additionally, our sensitivity analysis mitigated the effects of daily variation and emphasized long-term trends, resulting in *online doctor* and *online health* becoming significant. This is consistent with an earlier study that used weekly Google Trends data using the same set of keywords [15].

One strength of this study is the low probability of bias resulting from an anticipation effect leading up to the WHO pandemic declaration. Based on previous analyses of airline traffic [53] and Google Mobility data [54], as well as health-related Google searches [55], substantial changes to these patterns were observed only after the official WHO declaration was announced. For example, airline traffic remained constant until the week of 9 March 2020 [53]. Likewise, daily Google Mobility data reported for the countries under study remained constant relative to a baseline period (i.e., 3 January through 6 February 2020) until time points after 11 March 2020 [54]. Furthermore, previous research on public interest in digital health during the pandemic found no structural breaks in the time-series beyond the pandemic announcement between February 2019 and August 2020 [15]. In contrast, previous research using Google Trends data that used the implementation of lockdowns as cut-off point for their difference-in-differences designs reported the presence of an anticipation effect that started between one and three weeks prior to the start of the lockdown [24].

Some limitations of this study need to be considered. Unfortunately, we were not able to collect information on sociodemographic characteristics. Thus, we cannot rule out the possibility of selection bias. This is because young people, women, individuals with higher levels of education and/or income, and individuals representing the racial/ethnic majority in their societies are more likely to seek health information online, possibly due to these groups being more digitally connected and skilled [6,56–58]. These findings also imply that the needs and desires of a substantial cohort of individuals (i.e., those who may be unable to make full use of digital resources [57]) will not be represented in Google Trends data. Furthermore, we recognize that we collected data from only one search engine. While Google remains the most popular search engine, other sources of information may be used by those seeking digital healthcare. Additionally, Google search queries in languages other than English (e.g., in Canada and the United States, where French and Spanish, respectively, are the dominant languages of a large percentage of the population) were not captured in this study. Similarly, while we captured a large number of possible keywords, it remains possible that some linguistic nuances pertaining to digital healthcare were not captured by the reviews underpinning our data collection [34–36]. For some search terms, we observed mild search volume increases prior to the pandemic announcement, also observed in other studies using Google Trends data to examine the effects of COVID-19 [24]. While we have used a methodologically robust and widely applied approach, some results should be interpreted with caution. Our study also does not capture digital health-seeking behavior of individuals who contacted a general practitioner or health resource and instead received a direct referral to digital services.

Our findings highlight the changes of search behaviors when populations were faced with disruptions in traditional health services. However, the ongoing use of digital health and its structural implementation will require additional transformative work. Digital health services must be recognized as a valid form of healthcare. Comprehensive policy frameworks should be developed that will guide the sustained implementation and reimbursement of digital health services.

## Supporting information

S1 Appendix Supplementary materials. (DOCX)
