# Supplementary material for: Changes in digital healthcare search behavior during the early months of the COVID-19 pandemic: A study of six English-speaking countries
Source: PLOS Digit Health. 2023 May 1;2(5):e0000241. doi: 10.1371/journal.pdig.0000241 (PMC10150970; doi:10.1371/journal.pdig.0000241)
Supplement: S1 Appendix — (DOCX) [file pdig.0000241.s001.docx]

**Supplementary Materials**

van Kessel, R., Kyriopoulos, I., Mastylak, A., Mossialos, E. Changes in digital healthcare search behavior during the early months of the COVID-19 pandemic: a study of six English-speaking countries

Table of Contents

[Figure A. Relative search volumes for Australia 3](#_Toc130450640)

[Figure B. Relative search volumes for Canada 3](#_Toc130450641)

[Figure C. Relative search volumes for New Zealand 4](#_Toc130450642)

[Figure D. Relative search volumes for United Kingdom 4](#_Toc130450643)

[Figure E. Relative search volumes for United States 5](#_Toc130450644)

[Figure F. Relative search volumes for Ireland 5](#_Toc130450645)

[Figure G. Heatmap highlighting what keywords showed significant changes per studied country. 6](#_Toc130450646)

[Table A. Common trends assumption test results using digital health search volumes from 2016 to 2018. 6](#_Toc130450647)

[Table B. Sensitivity analysis using 7-day moving averages of Google Trends search volumes. 6](#_Toc130450648)

# Figure A. Relative search volumes for Australia

# Figure B. Relative search volumes for Canada

# Figure C. Relative search volumes for New Zealand

# Figure D. Relative search volumes for United Kingdom

# Figure E. Relative search volumes for United States

# Figure F. Relative search volumes for Ireland

# Figure G. Heatmap highlighting what keywords showed significant changes per studied country.

|  | **Australia** | **Canada** | **New Zealand** |  |  | Significant increase |
| --- | --- | --- | --- | --- | --- | --- |
| Online Doctor |  |  |  |  |  | Significant decrease |
| Online Health |  |  |  |  |  | No significant change |
| Telehealth |  |  |  |  |  |  |
| Telemedicine |  |  |  |  |  |  |
| Health App |  |  |  |  |  |  |
|  | **United Kingdom** | **United States** | **Ireland** |  |  |  |
| Online Doctor |  |  |  |  |  |  |
| Online Health |  |  |  |  |  |  |
| Telehealth |  |  |  |  |  |  |
| Telemedicine |  |  |  |  |  |  |
| Health App |  |  |  |  |  |  |

# Table A. Common trends assumption test results using digital health search volumes from 2016 to 2018.

|  | Estimate | 95% CI | P-value | Observations |
| --- | --- | --- | --- | --- |
| **2016-2017** |  |  |  |  |
| Online doctor | -9.73 | -15.10 – -4.38 | < 0.001 | 2178 |
| Online health | -5.55 | -13.28 – 2.18 | 0.16 | 2178 |
| Telehealth | -0.66 | -6.75 – 5.44 | 0.83 | 2178 |
| Telemedicine | -3.13 | -7.51 – 1.23 | 0,16 | 2178 |
| Health app | -4.86 | -12.47 – 2.75 | 0.21 | 2178 |
| **2017-2018** |  |  |  |  |
| Online doctor | -3.00 | -7.54 – 1.54 | 0.19 | 2172 |
| Online health | -5.77 | -10.70 – -0.84 | 0.02 | 2172 |
| Telehealth | -4.43 | -10.80 – 1.93 | 0.17 | 2172 |
| Telemedicine | -1.24 | -5.21 – 2.73 | 0.54 | 2172 |
| Health app | 0.46 | -2.76 – 3.68 | 0.78 | 2172 |

# Table B. Sensitivity analysis using 7-day moving averages of Google Trends search volumes.

|  | Estimate | 95% CI | P-value | Observations |
| --- | --- | --- | --- | --- |
| Online doctor | 5.89 | 2.96 – 8.82 | < 0.001 | 2154 |
| Online health | 7.49 | 5.51 – 9.47 | < 0.001 | 2154 |
| Telehealth | 10.80 | 7.63 – 13.97 | < 0.001 | 2154 |
| Telemedicine | 9.24 | 5.57 – 12.90 | < 0.001 | 2154 |
| Health app | 8.57 | 6.05 – 11.09 | < 0.001 | 2154 |
